# MicroRNA and circular RNA profiling in the deposited fat tissue of Sunite sheep

**DOI:** 10.3389/fvets.2022.954882

**Published:** 2022-11-04

**Authors:** Xige He, Rihan Wu, Yueying Yun, Xia Qin, Yajuan Huang, Lu Chen, Yunfei Han, Jindi Wu, Lina Sha, Gerelt Borjigin

**Affiliations:** ^1^College of Food Science and Engineering, Inner Mongolia Agricultural University, Hohhot, China; ^2^College of Biochemistry and Engineering, Hohhot Vocational College, Hohhot, China; ^3^School of Life Science and Technology, Inner Mongolia University of Science and Technology, Baotou, China

**Keywords:** Sunite sheep, tail fat, microRNA, circular RNA, competing endogenous RNA

## Abstract

As the most typical deposited fat, tail fat is an important energy reservoir for sheep adapted to harsh environments and plays an important role as a raw material in daily life. However, the regulatory mechanisms of microRNA (miRNA) and circular RNA (circRNA) in tail fat development remain unclear. In this study, we characterized the miRNA and circRNA expression profiles in the tail fat of sheep at the ages of 6, 18, and 30 months. We identified 219 differentially expressed (DE) miRNAs (including 12 novel miRNAs), which exhibited a major tendency to be downregulated, and 198 DE circRNAs, which exhibited a tendency to be upregulated. Target gene prediction analysis was performed for the DE miRNAs. Functional analysis revealed that their target genes were mainly involved in cellular interactions, while the host genes of DE circRNAs were implicated in lipid and fatty acid metabolism. Subsequently, we established a competing endogenous RNA (ceRNA) network based on the negative regulatory relationship between miRNAs and target genes. The network revealed that upregulated miRNAs play a leading role in the development of tail fat. Finally, the ceRNA relationship network with oar-miR-27a_R-1 and oar-miR-29a as the core was validated, suggesting possible involvement of these interactions in tail fat development. In summary, DE miRNAs were negatively correlated with DE circRNAs during sheep tail fat development. The multiple ceRNA regulatory network dominated by upregulated DE miRNAs may play a key role in this developmental process.

## Introduction

Adipose tissue is distributed in various parts of the sheep body and plays a crucial role in maintaining the balance of homeostatic metabolic processes in the body. Generally, adipose tissue can be found in the subcutaneous layer under the skin, around the kidneys, and within the abdominal cavity, and the tail, especially the tail fat is one of the most typical deposited fat. The “fat tail” trait of sheep is regarded as an adaptive response to the harsh environments and the fat stored in the tail is a valuable reserve for sheep during migration and in winter when food is scarce (1).

MicroRNAs (miRNAs) are single-stranded non-coding RNA molecules which are encoded by genes and bind to target mRNA transcripts *via* complementary base-pairing to exert their effects on expression (2). Several recent studies have analyzed sheep tail fat *via* miRNA-seq. The miRNA expression profiles of tail fat from Guangling large-tailed sheep and small-tailed Han sheep have been analyzed (2). A total of 40 differentially expressed (DE) conserved miRNAs were identified, in addition to 150 significantly expressed miRNAs, suggesting that these may play a role in the regulation of tail fat metabolism. Another study compared tail fat miRNA expression between Kazakhstan sheep (fat-tailed) and Tibetan sheep (thin-tailed) (3), revealing 539 miRNAs that were found in both breeds, of which 35 were novel. These miRNAs were involved in the MAPK, FoxO, and Wnt signaling pathways *via* their target mRNAs, thus influencing fat deposition and lipid metabolism in the fat tail. Current miRNA-seq studies have focused on other sheep tissues such as intramuscular fat (4), testis (5), and uterus (6), but studies related to tail adipose tissue are relatively lacking. Thus, the involvement of miRNAs in sheep tail fat metabolism requires further in-depth research. Different types of non-coding RNA perform a wide variety of biological functions and are involved in the regulation of diverse important pathways. These constitute endogenous RNA (ceRNA) networks, in which miRNAs play a central role. Recent studies have found that circular RNA (circRNAs) act as miRNA sponges, isolating miRNAs through competitive interactions with target mRNAs (7). CircRNAs are another class of non-coding RNAs, which are formed by covalent binding (reverse splicing) of the 5′ end of a linear RNA to the 3′ end (8). Since circRNAs have no free 5′ or 3′ end, they are not cleaved by exonucleases, which makes them more stable than most linear RNAs (9). A recent study in pig adipose tissue revealed that circRNA26852 and circRNA11897 target genes may be involved in adipocyte differentiation and lipid metabolism (10). Similarly, in buffalo, circRNAs 19:45387150|45389986 and 21:6969877|69753491 were shown to regulate fat deposition (11). In our previous study, we characterized the lncRNA and mRNA expression profiles of tail adipose tissue from Sunite sheep (SS) (12). The results showed that a total of 223 differentially expressed genes (DEGs) and 148 differentially expressed lncRNAs were found in tail fat, and the interaction of these genes may be involved in the metabolism of sheep tail fat. However, related research on miRNA and circRNA in SS tail fat is still lacking, including the regulation mechanism of fat metabolism.

SS is a Mongolian meat breed, and the distinctive phenotypic feature of SS is the fat tail. SS is a representative local superior breed within Inner Mongolia, with cold tolerance, drought resistance, rapid growth and development, high vitality, delicate flesh, and good flavor, which altogether make them very popular among consumers. The fat tail, of these sheep is the most typical deposited fat and helps SS adapt to harsh conditions such as cold, drought, and food shortages (13, 14), making them more adaptable than other breeds (15). The tail fat increases continuously with age, reaching a weight of ~3–4.5 kg at 30 months of age. As a by-product of mutton, tail fat is widely used as a raw material for the production of various daily necessities. Also, it can be an important source of dietary fat (1, 16), providing the human body with the energy it needs. While the deposition of tail fat in sheep may affect their intramuscular fat content to a certain extent (17), tail fat metabolic regulation is yet to be investigated. SS are mainly raised naturally grazing in the Sunite grassland of Inner Mongolia. These sheep are accustomed to autonomous activity and completely voluntary feeding. Thus, the deposition and metabolism of SS tail fat might be affected by a large number of potential factors (including feeding behavior, aging processes, pasture changes, seasonal alterations, and climate impact). In the present work, the expression profiles of miRNA and circRNA in deposited fat (tail fat) from SS at the ages of 6 months (6 M), 18 months (18 M), and 30 months (30 M) were analyzed. We sought to elucidate mechanisms underlying tail fat metabolism by constructing a ceRNA co-regulatory network, thus providing valuable insights into the transcriptome associated with sheep fat tissue metabolism and utilization of by-products of meat breeds of sheep.

## Materials and methods

### Sample collection

The experimental animals were nine castrated Sunite rams at 6 (6 M, *n* = 3), 18 (18 M, *n* = 3), and 30 months of age (30 M, *n* = 3). All sheep were raised under the same conditions (food, water source, and environment) in the Xilingol grassland. After slaughter (in October), adipose tissue from the tail fat (top 1/3) from each sheep was obtained and immediately frozen in liquid nitrogen.

### RNA extraction, sequencing, and transcript assembly

Total RNA was extracted from each sample using TRIzol reagent (Invitrogen, CA, USA) according to the manufacturer's instructions. The quantity and purity of total RNA were determined using a Bioanalyzer 2100 and RNA 6000 Nano LabChip Kit (Agilent, CA, USA), respectively. Approximately 1 μg of total RNA was used for small RNA library construction with TruSeq Small RNA Sample Prep Kits (Illumina, San Diego, USA), and single-end sequencing (36 or 50 bp) was performed on an Illumina HiSeq 2500. Subsequently, raw reads were subjected to ACGT101-miR (LC Sciences, Houston, TX, USA) to remove repeats, junk and low complexity, adapter dimers, and common RNA families (rRNA, tRNA, snRNA, and snoRNA). Then, unique sequences with lengths of 18–26 nucleotides were mapped to specific species precursors and the genome in miRBase 21.0 *via* BLAST search in order to identify known and novel miRNAs.

Approximately 10 μg of total RNA was used to deplete ribosomal RNA using the Epicenter Ribo-Zero Gold Kit (Illumina, San Diego, USA) as per manufacturer instructions. The remaining RNA fragments were then reverse-transcribed to form the final complementary DNA (cDNA) library using an RNA-seq Library Preparation Kit (Illumina, San Diego, USA) according to the manufacturer's protocol. Finally, paired-end sequencing on an Illumina HiSeq 4000 was performed following the manufacturer's protocol. Mapped reads were assembled into circRNA using CIRCExplorer (18, 19). Tophat-fusion and CIRCEexporer were used to identify the back-splicing reads among the unmapped reads.

### Differential expression analysis

The differential expression of miRNAs based on normalized deep-sequencing counts was analyzed using a *T*-test. The threshold for differential expression was set at *P* < 0.05. SRPBM was used to normalize the expression of circRNA in our study (20). Differentially expressed circRNAs were selected based on |log2 (fold change)| > 1 and *P* < 0.05 using the R package Ballgown (21). Further, we performed trend enrichment analysis on the DE miRNAs using the Short Time-series Expression Miner (STEM) software (22) as well as significant enrichment analysis with a threshold of *P* < 0.05.

### Target gene prediction and functional analysis

To explore the functions of DE miRNAs, circRNAs and mRNAs were predicted as miRNA targets using TargetScan (23) and miRanda (24). A TargetScan Score > 50 and miRanda Energy < −20 were considered indicative of a targeting relationship. We then performed GO and KEGG analyses of DE miRNA targets and the host genes of DE circRNAs using in-house scripts. Statistical significance was set at *P* < 0.05. SS tail fat mRNA (including circRNA) and miRNA data were deposited in the NCBI Sequence Read Archive (SRA) database under accession numbers PRJNA791005 and PRJNA790717, respectively.

### Construction of ceRNA co-expression network

After determining the target relationships of DE miRNAs using TargetScan and miRanda, we screened miRNA-targets with negative regulatory relationships (e.g., down regulation-up regulation) from these target relationships to build the mRNA-miRNA-circRNA co-expression networks. DE mRNAs were filtered based on *P* < 0.05 and |log2 (fold change)| > 1. Cytoscape (version 3.9.0) was used to visualize the network.

### qRT-PCR validation

In our study, six DE miRNAs and eight DE circRNAs were randomly selected to validate RNA-seq data using real-time quantitative PCR. The expression of each miRNA and circRNA was calculated *via* the 2^−ΔΔCT^ method, with GAPDH (25, 26) and U6 used as reference genes for circRNA and miRNA, respectively. The primer information is shown in the Supplementary Table S1.

### Dual-luciferase gene reporter analysis

The psiCHECK2-target WT (wild type) gene was synthesized by inserting a target gene fragment containing the miRNA-binding sequence into the luciferase gene of the psiCHECK-2 vector. The mutant vector pCK TCP1-M was created by mutating the miRNA-binding sites using overlapping extension PCR. HEK293T cells were seeded into 24-well plates at a density of 1 × 10^5^ cells/well and incubated at 37°C overnight. The miRNA mimics, psiCHECK2-target WT gene, and psiCHECK2-target MUT (mutant traits) gene were transfected into cells. At 48 h post-transfection, the Renilla luciferase activity/firefly luciferase activity was determined using the dual-luciferase reporter gene assay system (Promega).

## Results

### Summary of RNA-seq analysis

**Figure 1:**
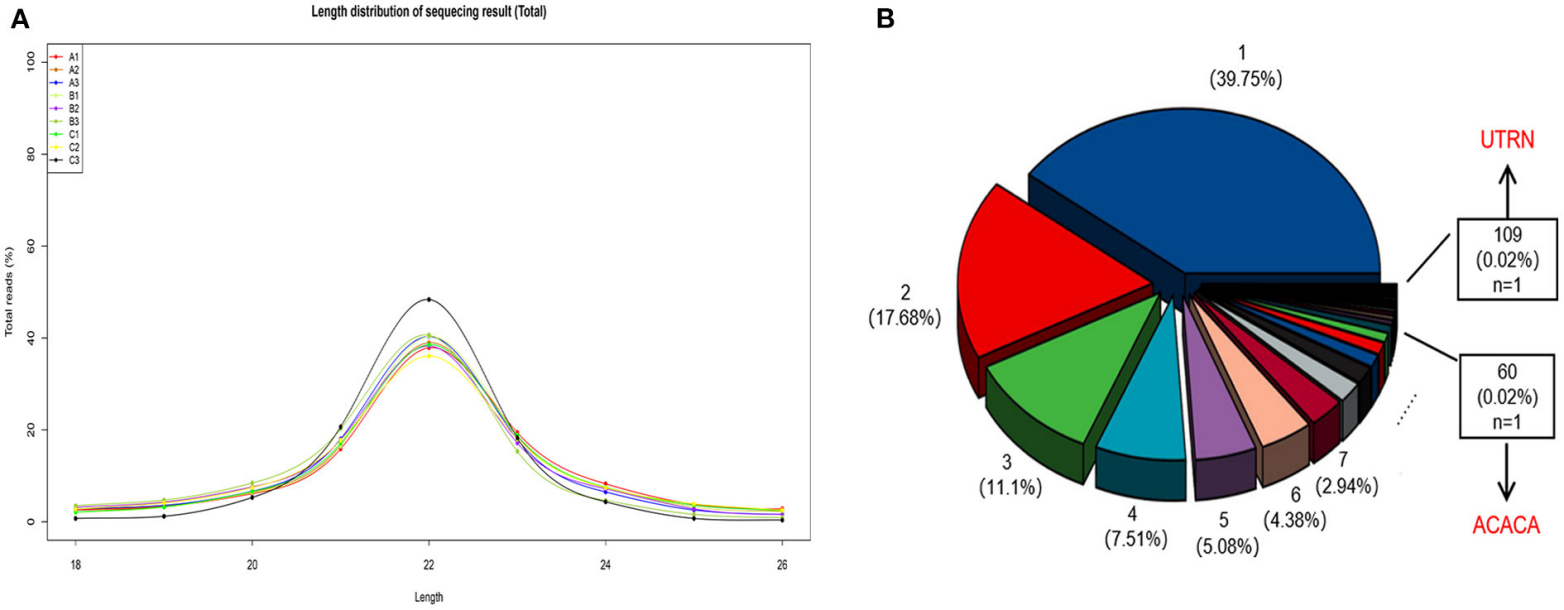
**(A)** The length distribution of sequenced miRNAs. 6M: A1, A2, A3; 18M: B1, B2, B3; 30M: C1, C2, C3. **(B)** Percentage of circRNA host genes. For example, the blue pie chart shows that 39.75% of the genes are transcribed to form 1 circRNA, meanwhile the red pie chart shows that 17.68% of the genes can be transcribed to make 2 circRNAs, and so on.

We obtained 1,942 miRNAs, including 392 novel miRNAs, and the majority of reads were ~21–23 nucleotides (nt) in length, which corresponds to the typical length following Dicer enzyme cleavage (Figure 1A). We also identified 17,531 circRNAs and 4,767 host genes. As shown in Figure 1B, 39.75% of the circRNAs were transcribed from a unique mRNA. UTRN (0.02%) was the most common host gene, giving rise to 109 circRNAs. Researchers have shown that the UTRN gene is related to pig intramuscular fat (27). In addition, ACACA, which plays a key role in the regulation of fatty acid synthesis, was a host gene of 60 circRNAs. Thus, these circRNAs may exert a potential regulatory effect on fat metabolism in sheep tails by modulating host gene expression.

### Differential expression analysis

**Figure 2:**
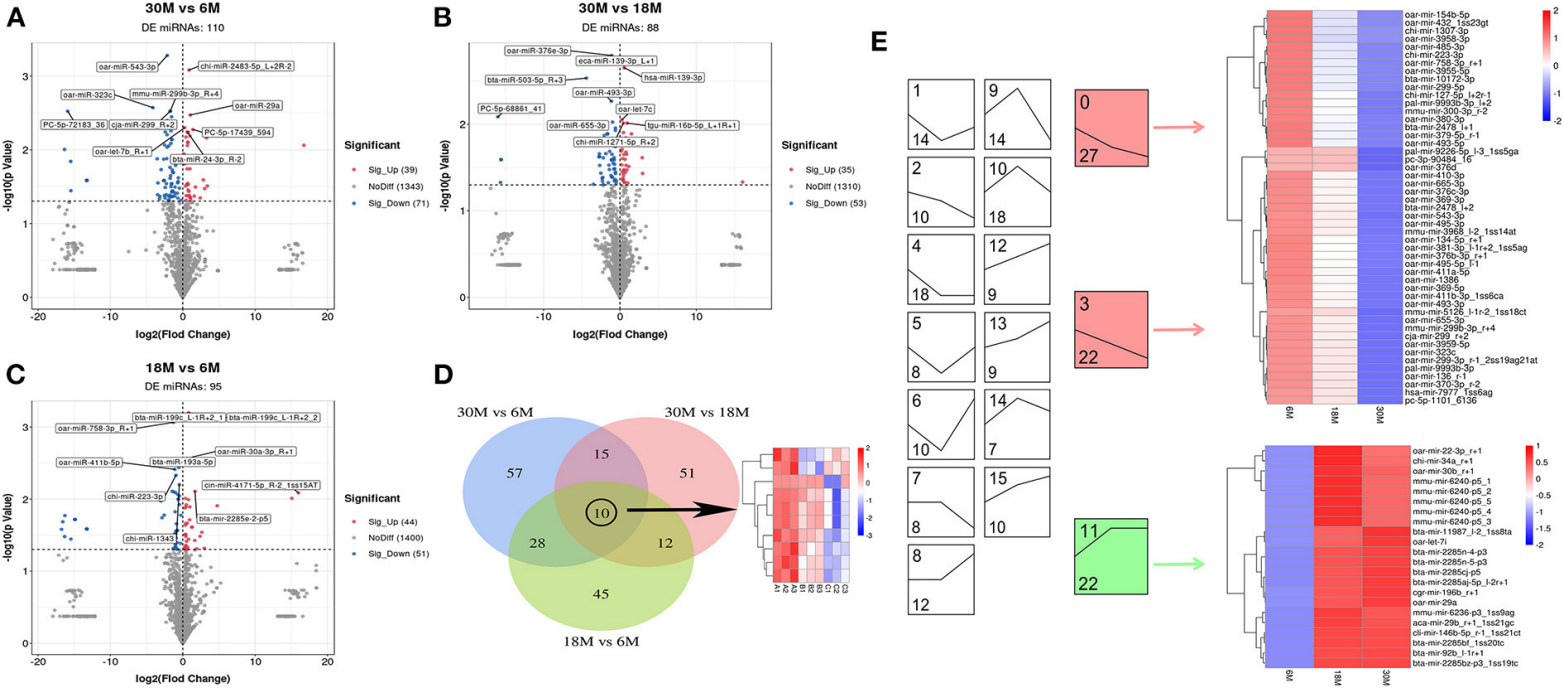
miRNA differential expression analysis. **(A–C)** The volcano plot of DE miRNA. **(A)** 30M vs. 6M; **(B)** 30M vs. 18M; **(C)** 18M vs. 6M. Annotated as the top five DE miRNA based on *P*-value. **(D)** Venn diagram analysis of DE miRNA. **(E)** Expression trend analysis of DE miRNA. The upper number indicates the ordinal number of each trend, while the lower number is the number of genes enriched, and those with color are the significantly enriched trends.

We compared miRNAs (Supplementary Table S2) and circRNAs (Supplementary Table S3) in the tail fat of SS at three different stages (30 vs. 6 M, 30 vs. 18 M, and 18 vs. 6 M). The largest number of DE miRNAs were obtained for the 30 vs. 6 M comparison, with a total of 110 DE miRNAs (39 upregulated and 71 downregulated), including four novel DE miRNAs (Figures 2A–C). The 30 vs. 18 M comparison yielded 88 DE miRNAs (35 upregulated and 53 downregulated), including 3 novel DE miRNAs. For 18 vs. 6 M, 95 DE miRNAs (44 upregulated and 51 downregulated) were obtained, including seven novel DE miRNAs. Furthermore, 10 overlapping DE miRNAs were identified between the three comparison groups. These were highly expressed in 6 M SS, and their expression decreased with age (Figure 2D). Therefore, we postulated that these DE miRNAs may be downregulated in parallel to the increase in tail fat, validating this hypothesis *via* trend enrichment analysis and heatmaps (Figure 2E). The DE miRNAs were enriched in 16 terms, three of which were significantly enriched (*P* < 0.05), colored in red and green. Forty-nine DE miRNAs were enriched with downregulated trends (red), and 22 DE miRNAs were enriched with upregulated trends (green), indicating that the DE miRNAs were mainly downregulated, which confirmed our hypothesis.

**Figure 3:**
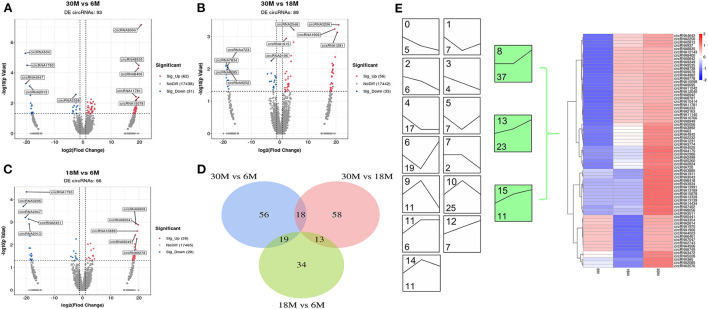
circRNA differential expression analysis. **(A–C)** Volcano plot of DE circRNAs. **(A)** 30M vs. 6M; **(B)** 30M vs. 18M; **(C)** 18M vs. 6M. Annotated as the top five DE circRNAs based on *P*-value. **(D)** Venn diagram analysis of DE circRNA. **(E)** DE circRNA expression trend analysis. The upper number indicates the ordinal number of each trend, while the lower number is the number of genes enriched, and those with color are the significantly enriched trends.

A total of 93 (62 upregulated and 31 downregulated), 89 (56 upregulated and 33 downregulated), and 66 (38 upregulated and 28 downregulated) DE circRNAs were obtained for 30 vs. 6 M, 30 vs. 18 M, and 18 vs. 6 M, respectively (Figures 3A–C). None of these were found to overlap among the three comparison groups (Figure 3D). Compared with DE miRNA, the expression trends in DE circRNAs were mainly enriched in the upregulation trend (green), indicating that these DE circRNAs may play a key role in the later stages of sheep fat tail growth (Figure 3E). Overall, the majority of DE miRNAs and circRNAs exhibited contrasting expression, indicating that the negative regulation between these molecules may play an important role in SS tail fat development.

### Target gene prediction analysis

To build a ceRNA network of mRNA-miRNA-circRNA in sheep tail fat, miRNA target genes were predicted. To this end, we used TargetScan and miRanda, and all DE miRNAs (110) from the 30 vs. 6 M comparison were shown to bind to at least one mRNA. Four DE miRNAs were bound to more than 100 mRNAs. chi-miR-1343 targeted the most mRNAs (118), followed by oar-miR-370-3p_R-2 (111), bta-miR-2387_R+1 (102), and oan-miR-103-3p_R+2 (100). Among 30 vs. 18 M and 18 vs. 6 M, the novel PC-3p-43105_133 was the only DE miRNA targeting more than 100 mRNAs, 109 mRNAs, and 103 mRNAs, respectively. Thus, it could be a major regulator of fat tail development. Likewise, chi-miR-1343 was predicted to bind 88 mRNAs in 30 vs. 18 M and 83 mRNA in the 18 vs. 6 M comparison, suggestive of a multifaceted regulatory role.

In contrast, we predicted a binding interaction between DE miRNAs and DE circRNAs. For 30 vs. 6 M, there were 81 circRNAs bound to 99 miRNAs. Nine circRNAs were predicted to bind oar-miR-27a_R-1 and chi-miR-27b-3p. circRNA9695 was bound to the largest number of miRNAs (24), and three other circRNAs were bound to more than 10 miRNAs, namely, circRNA4175 (16), circRNA1985 (11), and circRNA382 (11). Likewise, these three circRNAs were found to bind multiple miRNAs from the 30 vs. 18 M comparison group. Thus, circRNA4175, circRNA1985, and circRNA382 could be major regulators of fat tail development. In 30 vs. 18 M, we found 71 circRNA and 71 miRNA targeting relationships, and the novel miRNA PC-3p-43105_133 was bound to the most circRNAs (13), once again highlighting the importance of this comparison group. Another miRNA family, miR-16a (chi-miR-16a-5p and oar-miR-16b_R+3), was found to bind to nine circRNAs. The 65 miRNAs were bound to 50 circRNAs in 18 vs. 6 M. mmu-miR-6240-p5 was found to bind the most mRNAs (9). Taken together, we predicted that multiple miRNAs bind to circRNAs that may play a key role in SS tail fat growth.

### Validation of sequencing results

**Figure 4:**
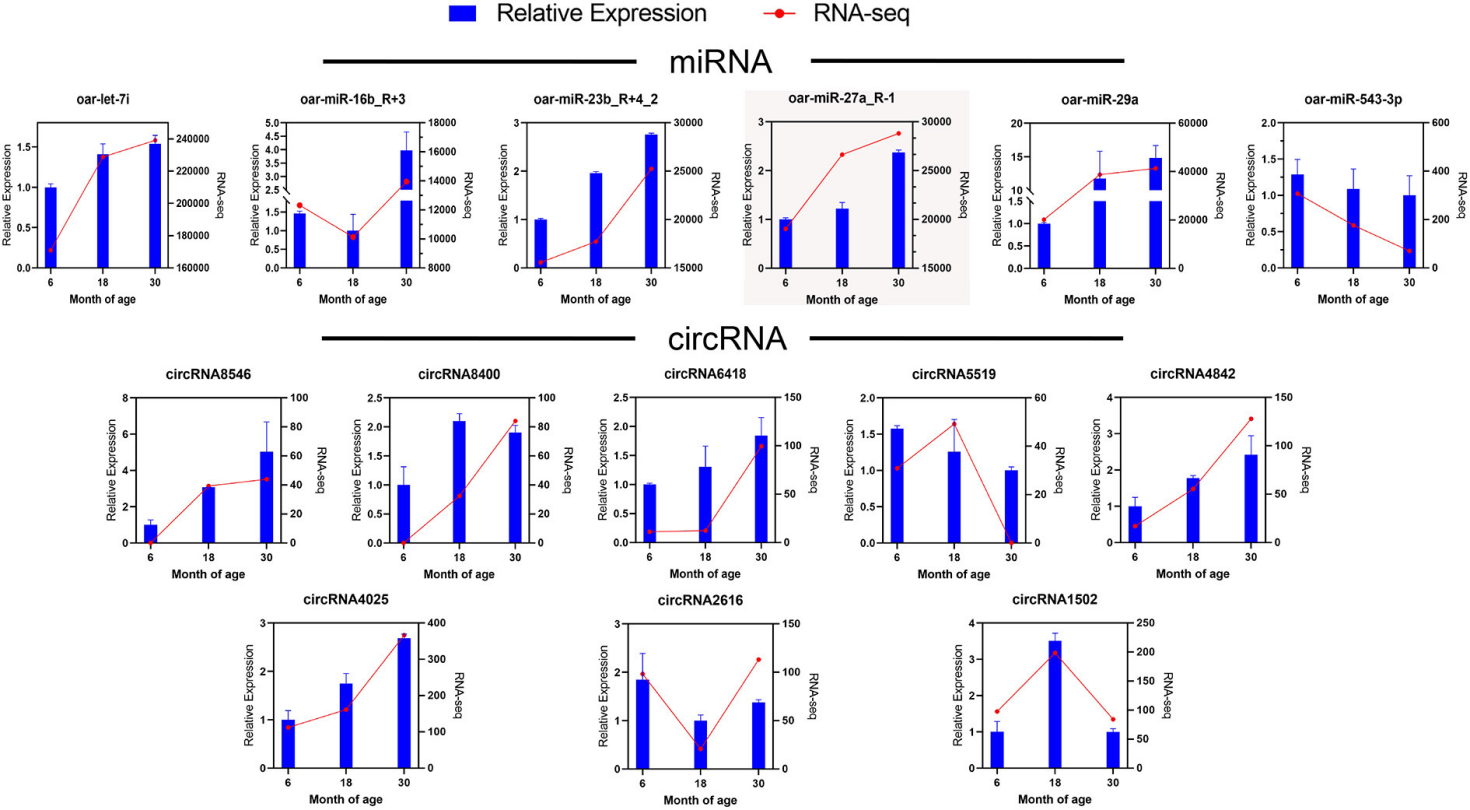
Validation of DE miRNAs and DE circRNAs *via* qRT-PCR. The blue bar represents qRT-PCR data, and the red line represents RNA-seq data.

To validate RNA-seq results, we randomly selected six miRNAs and eight circRNAs and measured their expression in the 6, 18, and 30 M groups *via* qRT-PCR (Figure 4). The relative expression data determined *via* qRT-PCR were consistent with FPKM values obtained *via* RNA-seq, indicating that the RNA-seq data were reliable.

### GO annotation and KEGG pathway analysis of miRNA target genes

**Figure 5:**
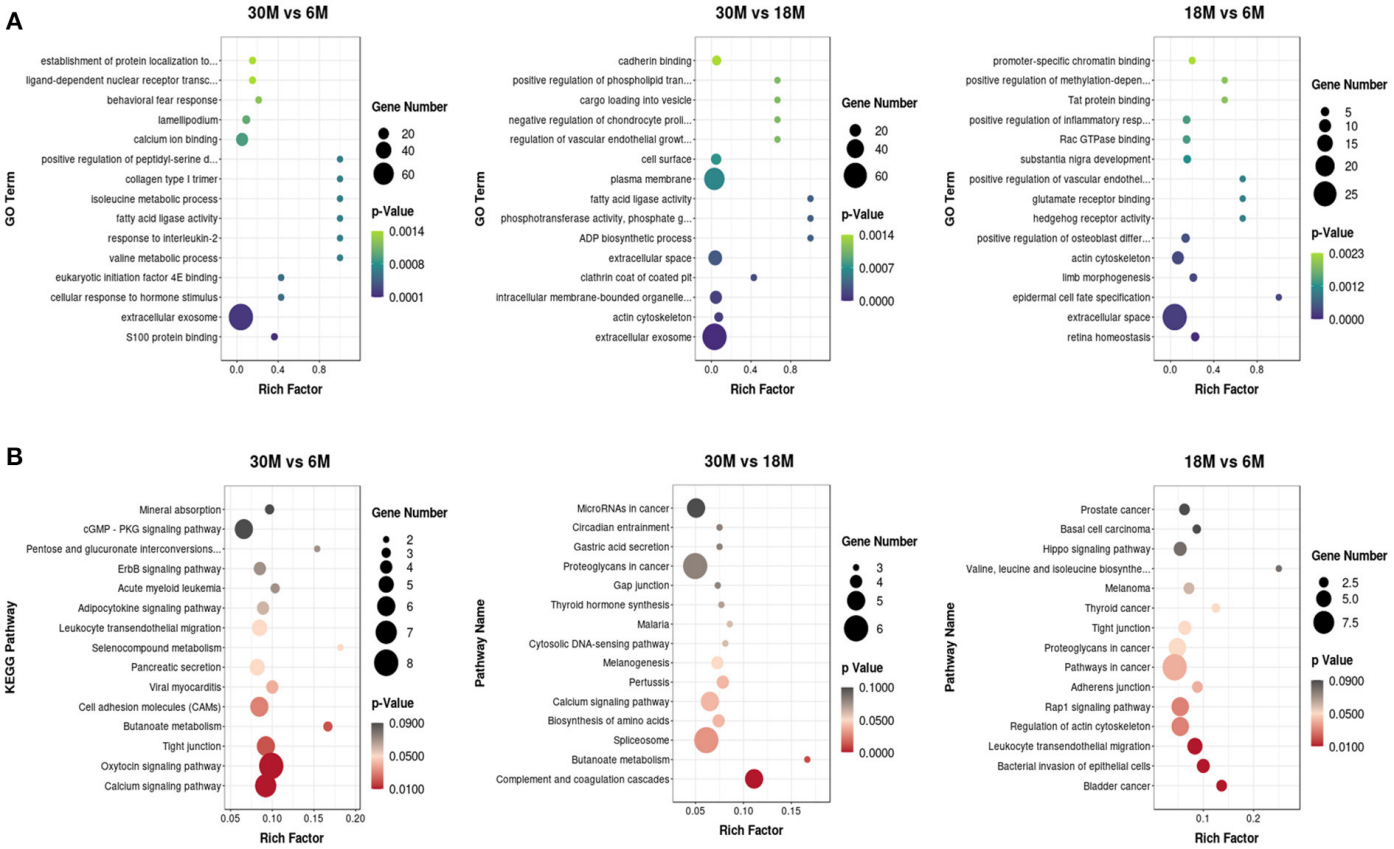
The top 15 GO and KEGG terms for DE miRNAs from the three comparison groups. **(A)** GO analysis; **(B)** KEGG analysis.

The GO enrichment analysis of target genes was classified into biological process (BP), cellular component (CC), and molecular function (MF) categories (Supplementary Figure S1). More than half of the enriched GO terms for the three comparison groups were from the BP category, including the lowest number. CC terms were enriched with the highest number of genes in comparison groups, which mainly included the membrane, nucleus, and exosomes. Taken together, tail fat development is driven by diverse biological processes and cellular interactions. In three different stages, the miRNA target genes were significantly enriched for 843 GO terms, and the top 15 most significantly enriched GO terms are presented in a scatter plot (Figure 5A). Among the three comparison groups, we identified five GO terms that were enriched between two groups. These included actin cytoskeleton, intracellular membrane-bound organelles, extracellular space, extracellular exosome, and focal adhesion of the CC category, in addition to fatty acid ligase activity of the MF category. These miRNAs may play a significant regulatory role in fatty acid metabolism.

KEGG pathway analysis of the miRNA target genes was also performed, and the top 15 KEGG pathways are shown in a scatter plot (Figure 5B). A total of 19 KEGG pathways were significantly enriched. Among these, we noticed that the Rap1 signaling pathway, adherens junction, tight junction, cell adhesion molecules (CAMs), and regulation of actin cytoskeleton were enriched in the different growth stages. Furthermore, butanoate metabolism was enriched in both the 30 vs. 6 M and 30 vs. 18 M comparison groups.

### Functional analysis of circRNA host genes

**Figure 6:**
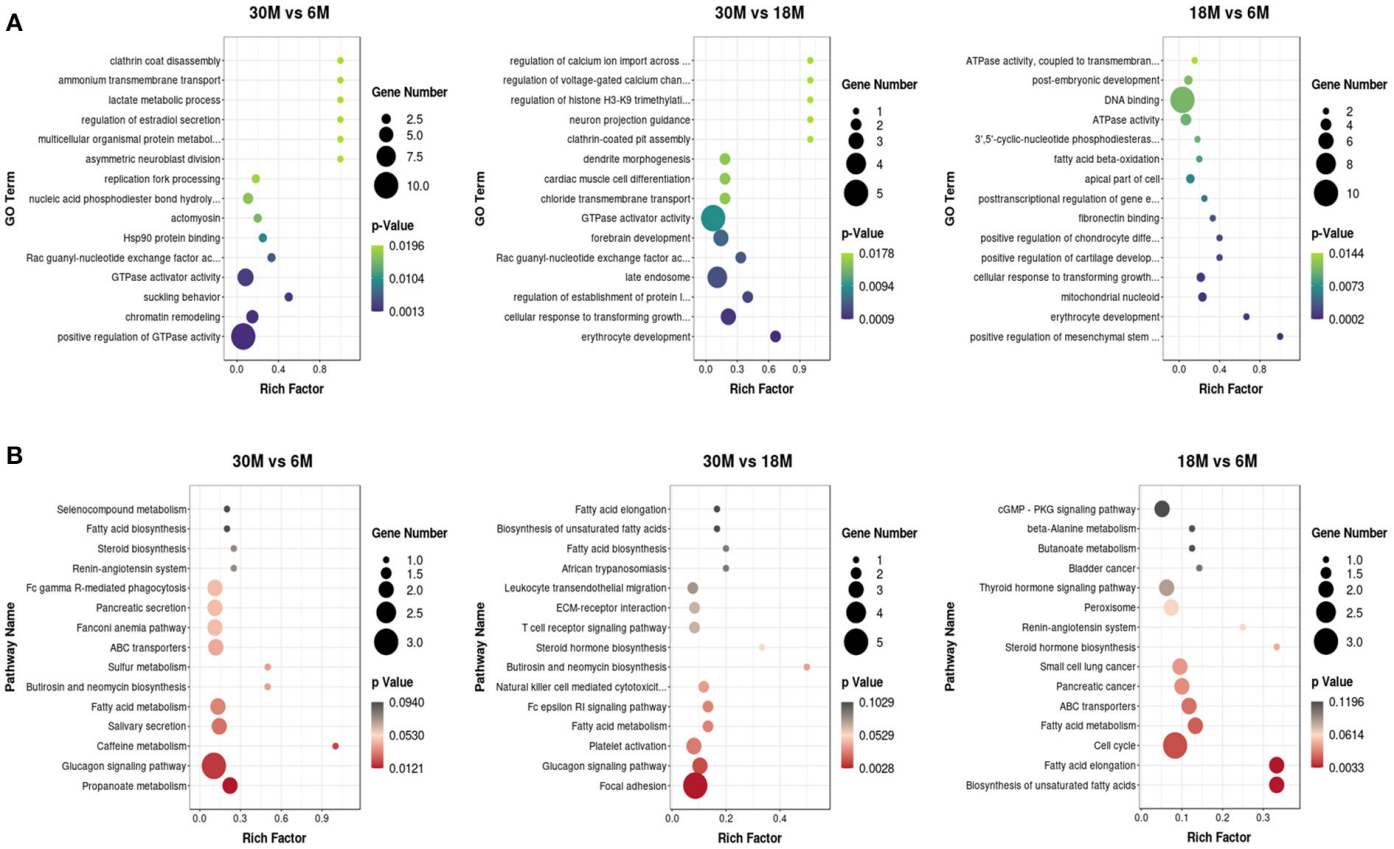
The top 15 enriched GO and KEGG terms for DE circRNAs. **(A)** GO analysis; **(B)** KEGG analysis.

We employed GO and KEGG enrichment analysis to investigate the role of circRNAs in tail fat growth mediated *via* their host genes. Most enriched GO terms belonged to the BP category, and most of the genes were enriched in CC (Supplementary Figure S2). We obtained 350 significantly enriched GO terms in the three comparison groups. The top 15 GO terms are presented in a scatter plot (Figure 6A). GTPase activator activity was enriched in both the 30 vs. 6 M and 30 vs. 18 M comparison groups, and fatty acid beta-oxidation was among the significantly enriched GO terms for 18 vs. 6 M. The top 15 enriched KEGG pathways are shown in Figure 6B. There were 22 significantly enriched KEGG pathways, among which several fat-related pathways were identified. These processes included propanoate metabolism, fatty acid metabolism, fatty acid biosynthesis, unsaturated fatty acid biosynthesis, and fatty acid elongation. Fatty acid metabolism was the only pathway enriched in all three groups. Host genes ACACA (circRNA382, circRNA392, and circRNA394) and HADHA (circRNA10888) were enriched in these pathways, indicative of the involvement of these circRNAs in fatty acid metabolism within tail fat.

### Construction of the mRNA-miRNA-circRNA co-expression network

**Figure 7:**
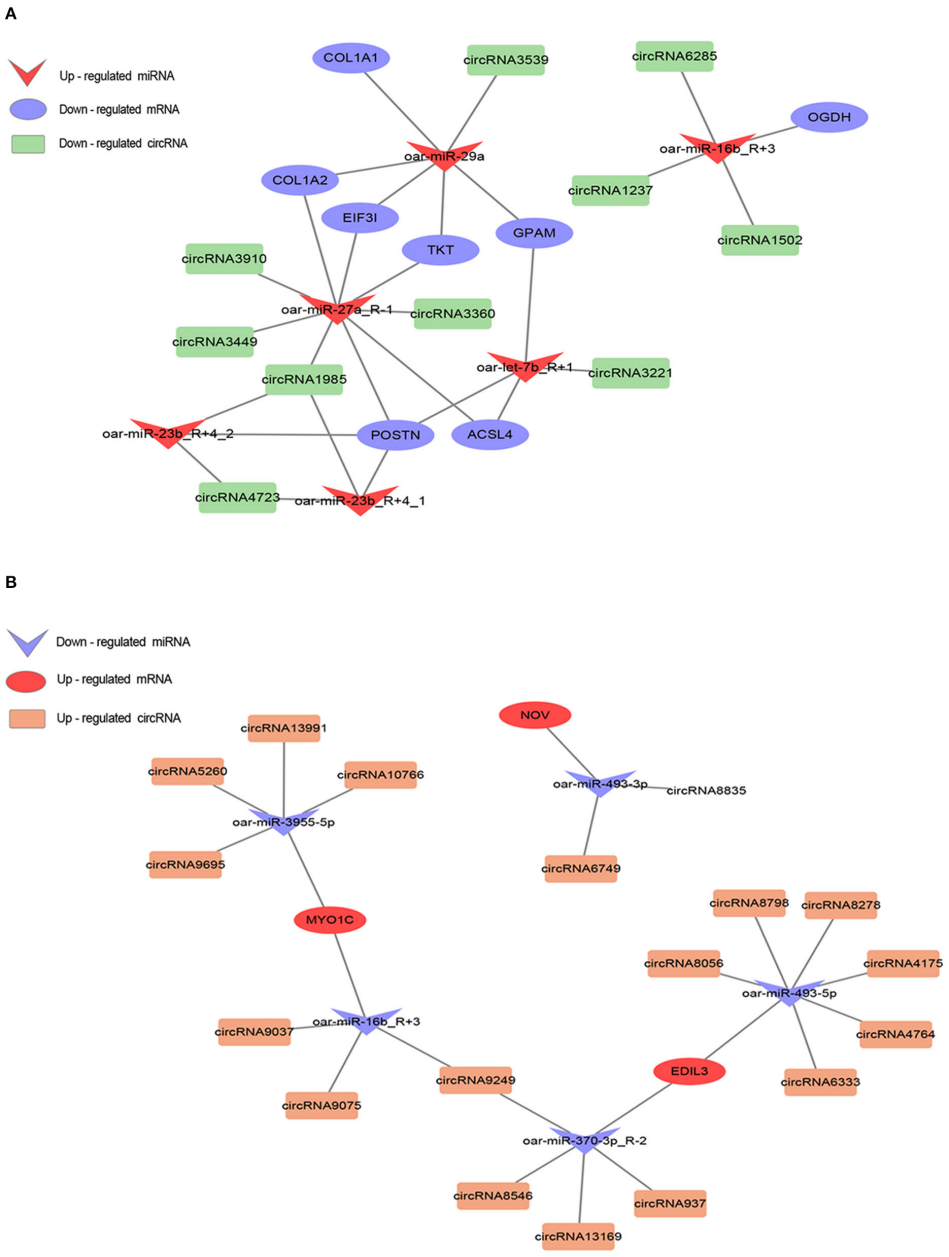
ceRNA regulatory network analysis in sheep tail fat. **(A)** Down-up-down mode. **(B)** Up-down-up mode. The shapes represent different RNAs and the colors represent different regulations.

Using Cytoscape (version 3.9.0), we constructed co-expression networks based on the mRNAs, miRNAs, and circRNAs identified in sheep fat tails (Figure 7). In this manner, we determined co-expression relationships based on the negative correlation between miRNA expression and target gene expression, given the targeting interactions between miRNA (sheep species), mRNA, and circRNA. A total of 35 (down-up-down) and 19 (up-down-up) mRNA-miRNA-circRNA co-expression patterns were obtained. This indicates that the down-up-down co-expression predominated the network, suggesting that upregulated miRNAs in the ceRNA network play a central regulatory role in tail fat. It is worth noting that oar-miR-16b_R+3 was targeted to two mRNAs and seven circRNAs, indicating that oar-miR-16b_R+3 also has regulatory functions with varied expression patterns at different stages of tail fat development. More importantly, we discovered that oar-miR-27a_R-1 and oar-miR-29a are at the center of the regulatory network and bind to several mRNAs and circRNAs, including TKT, ACSL4, GPAM, and POSTN. Among them, GPAM and ACSL4 are closely related to fat metabolism. Therefore, we suggest that oar-miR-27a_R-1 and oar-miR-29a have an important significance in the overall ceRNA relationship, and play a key role in the growth and metabolism of sheep tail fat.

### miRNA target validation

In our study, the dual-luciferase reporter system was used to verify the relationship between miRNAs and their targets. We further analyzed sheep DE miRNAs and their targets to identify ceRNAs that may be associated with tail fat growth. We filtered highly expressed sheep miRNAs (average norm value > 1,000) and sought to identify miRNAs that had a major effect on sheep tail fat development (Supplementary Table S4). Among these highly expressed miRNAs, oar-miR-27a_R-1 and oar-miR-29a were included, and the analysis above revealed that these genes were critical for the growth and metabolism of sheep tail fat. This led us to select these two miRNAs as the validation miRNAs. According to the ceRNA co-expression network, oar-miR-27a_R-1 was upregulated at 30 vs. 6 M and targeted to ACSL4, which is related to fatty acid metabolism. circRNA1985 was downregulated at 30 vs. 6 M, binding to oar-miR-27a_R-1. oar-miR-29a was upregulated at 30 vs. 6 M and 18 vs. 6 M, binding with 5 DEGs. Among them, GPAM was related to glycerolipid and glycerophospholipid metabolism. As a competitive binding RNA, circRNA3539 was downregulated in this process. Therefore, we sought to validate the ceRNA regulatory relationship between oar-miR-27a_R-1 and oar-miR-29a as the core, that is, between ACSL-oar-miR-27a_R-1-circRNA1985 and GPAM-oar-miR-29a-circRNA3539.

**Figure 8:**
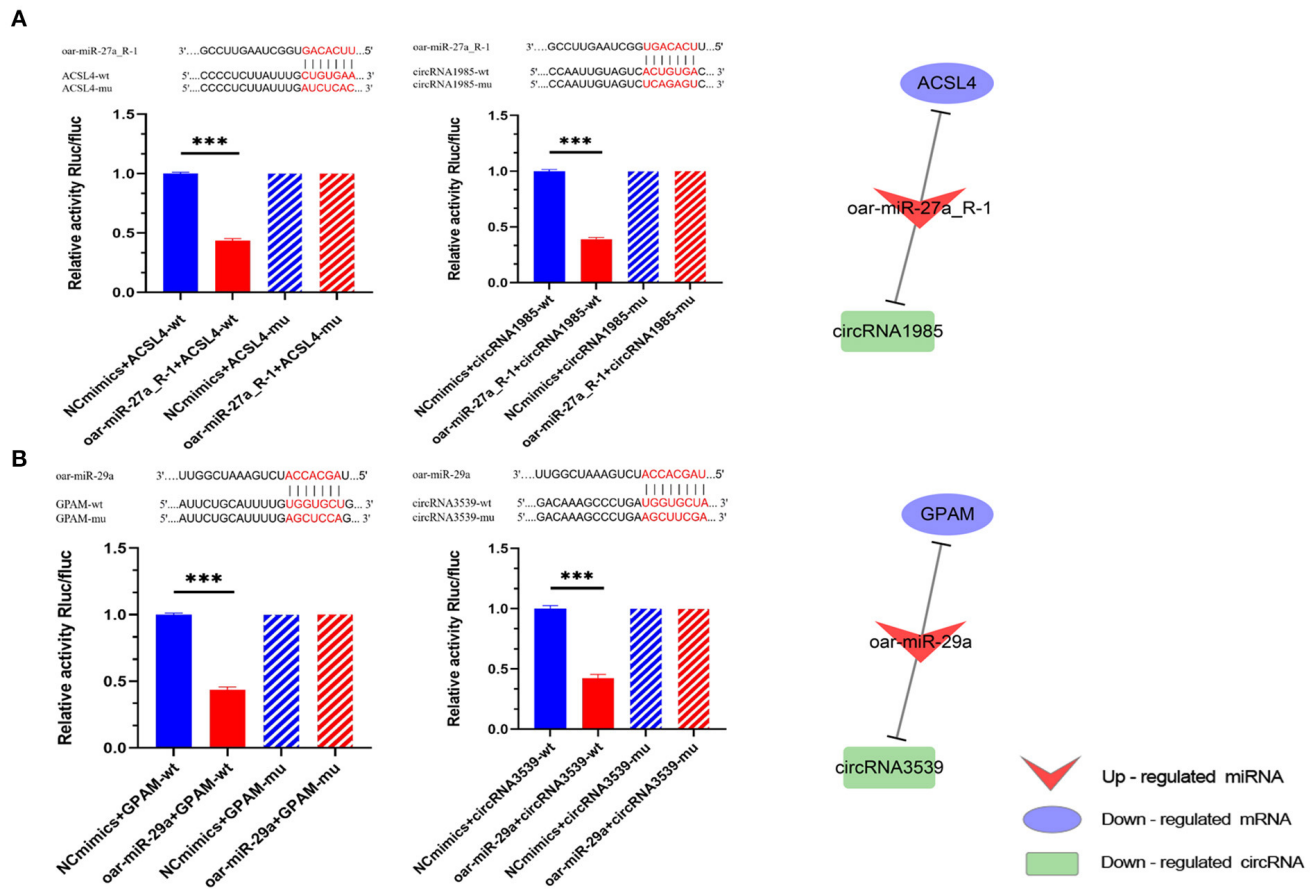
ceRNA dual-luciferase reporter gene analysis. **(A)** Binding site validation for oar-miR-27a_R-1 and ACSL4 as well as oar-27a_R-1 for circRNA1985; **(B)** Binding site validation for oar-miR-29a and GPAM as well as oar-miR-29a and circRNA3539. Bars without slashes show the result of inserting the mutant sequence into the plasmid, while solid bars show the result of inserting the original sequence. The group with extra miRNA sequences is shown in red, and the group without any miRNA sequences (control group) is shown in blue. ***P < 0.001.

We found only one target site in all the targeted pairs (Figure 8). Furthermore, compared with the negative control (NC) group, oar-miR-27a_R-1 and oar-miR-29a significantly downregulated the expression of luciferase in their predicted WT (wild type) targets (*P* < 0.001), indicating that there is a binding effect between all predicted targets in our study. After mutation, these three miRNAs failed to downregulate the expression of luciferase of mutant targets (*P* > 0.05) compared with the NC group, indicating that mutation was successful. Taken together, these results suggest that oar-miR-27a_R-1 can decrease ACSL4 expression by targeting the ACSL4-3′-UTR, and circRNA1985 can competitively bind with oar-miR-27a_R-1 and thus regulate the expression of ACSL4. The same conclusion was drawn for GPAM-oar-miR-29a-circRNA3539.

## Discussion

As a part of the sheep tissue, the tail fat can store heat for the sheep's body and help them resist the cold winter. As a by-product of mutton, tail fat provides the energy needed by the human body, and it can also be used as a raw material for human daily necessities, such as soap and medicinal materials. At present, sheep tail fat is gradually entering the field of vision of researchers. Previous studies have conducted transcriptome analysis on different breeds of sheep tails, and the results may be affected by the breed effect to some extent (26). However, the amount of intramuscular fat has been shown in previous study to be significantly impacted by aging (28). Generally, Sunite fat-tailed sheep mainly start slaughter at the age of 6 months, and after growth and development throughout the year, the tail fat increases continuously and reaches the weight of about 3–4.5 kg at 30 months of age. Therefore, in our study, we used the fat tail from Sunite sheep in three different stages, namely 6, 18, and 30 months to understand the potential molecular mechanism in fat development. The miRNAs and circRNAs were obtained by RNA-seq from the fat tail tissues of the three groups. Then, we explored the potential mechanism of sheep tail fat regulation in three different stages through functional analysis, and finally highlighted ceRNA regulation by the construction of co-expression networks and miRNA target validation.

With the advancement of RNA deep sequencing technology, miRNA-seq technology is increasingly being used in various animal species, resulting in the discovery of a large number of novel miRNAs. In this study, miRNAs from closely similar species found in miRBase were compared and identified during the analysis in order to more comprehensively refer to the registration data of miRBase. In spite of this, out of 1,942 miRNAs, we still found 392 novel miRNAs. The identification of these novel miRNAs may inspire new avenues of research in related areas, and additional research may provide insight on their biological significance in regulating lipid metabolism in sheep tail adipose tissue.

A total of 219 DE miRNAs (including 12 novel miRNAs) were detected in three comparison groups of different growth stages in sheep fat tail. Among these DE miRNAs, each comparison group had DE miRNAs that are uniquely expressed, and there were DE miRNAs that are commonly expressed in any two comparison groups or in three groups. We found that these DE miRNAs were mainly down-regulated and that their expression decreased with sheep age, which was supported by our expression trend enrichment analysis. Previous studies in Han sheep adipose tissue yielded similar results, and the mechanism underlying their down-regulation remains unknown (29).

CeRNAs regulate mRNA expression *via* competitively binding to miRNAs (30). We used TargetScan and miRanda to predict miRNAs target genes, and results showed that 110 DE miRNAs were predicted to bind to at least one mRNA. Among them, 5 DE miRNA were bound to more than 100 mRNAs, namely oar-miR-370-3p_R-2, bta-miR-2387_R+1, oan-miR-103-3p_R+2, chi-miR-1343, and PC-3p-43105_133. A previous study indicated that miRNA-1343-5p was predicted to bind to the key adipogenic gene C/EBP in bovine adipocytes (31). *In vivo* and *in vitro* studies suggest that miR-370 may alter fatty acid composition during adipogenesis, promote 3T3-L1 preadipocyte proliferation, and inhibit differentiation by directly targeting Mknk1 (32). Further, miR-370 may promote the expression of lipogenic genes SREBP-1c, DGAT2, FAS, and ACC1 (33). miR-103 has been described as playing a critical role in lipid metabolism. Studies have shown that miR-103 inhibits the expression of FASN and SCD1 *via* direct binding, in addition to promoting the differentiation of 3T3-L1 cells by targeting MEF2D and activating the AKT/mTOR signaling pathway (34, 35). miR-1343 may play a key role in sheep intestinal tissue based on PIK3R1 being its predicted target gene (36). In addition, chi-miR-1343 was also found to bind to multiple mRNAs in the 30 vs. 18 M and 18 vs. 6 M groups. Thus, we suggest that chi-miR-1343 may play an important regulatory role in sheep tail fat growth and development, and its mechanism in metabolic regulation needs to be further elucidated. Further, the novel PC-3p-43105_133 was the only DE miRNA targeting more than 100 mRNAs among the 30 vs. 18 M and 18 vs. 6 M groups. The novel miRNAs might represent a crucial regulatory component. For instance, recent research has demonstrated that the novel miRNA Y-56 targets IGF-1R to regulate the proliferation and cell cycle processes of porcine skeletal muscle satellite cells (37). However, there is still a limitation of novel miRNAs related to adipose tissue, and the mechanisms of many novel miRNAs remain unknown. As a result, it is essential to investigate the mechanisms of these novel miRNAs.

To uncover the functions of these DE miRNAs in tail adipose tissue, we utilized GO enrichment and KEGG pathway analyses. Among the three comparison groups, we found that GO functions of the actin cytoskeleton, intracellular membrane-bounded organelles, extracellular space, extracellular exosome, and focal adhesion of the CC category, and fatty acid ligase activity of the MF category, were enriched between two groups. Studies have shown that the inhibition of focal adhesion kinase (FAK) leads to an upregulation of adipogenic marker genes AP2 and LEP and lipid accumulation (38). The FATP family of proteins, which have fatty acid acyl-CoA ligase activity, is present in the plasma membrane and intracellular organelles (39). However, none of the miRNAs were found to bind to any of the genes in the FATP family in our study. Instead, ACSM1 and ACSM3, which are related to fatty acid ligase activity (http://geneontology.org/), were found to bind 24 miRNAs. These miRNAs may play a significant regulatory role in fatty acid metabolism. We also found several significant pathways in the KEGG pathway analysis, including the Rap1 signaling pathway, adherens junction, tight junction, cell adhesion molecules (CAMs), regulation of actin cytoskeleton, and butanoate metabolism. Tight junctions and adherens junctions are two types of cellular junctions that fundamentally affect cell proliferation and differentiation (40). Actin is a major component of adherens junctions, and abnormalities in this cytoskeletal protein impede the assembly of adherens junctions (41). Rap1 is mainly implicated in the control of cell adhesion, cell junction formation, secretion, and cell polarity (42). The significant enrichment of these pathways suggests that DE miRNAs may play prominent roles in sheep tail adipogenesis through cell-to-cell interactions. In addition, no studies have suggested a role for the Rap1 signaling pathway in adipose tissue. The present study may provide a theoretical basis for future research in this direction. Butyrate is processed by acetyl-CoA to generate fatty acids, cholesterol, and ketone bodies, thus providing specialized substrates for lipid biosynthesis (43). Butyrate treatment can cause adipocytes to accumulate triglycerides *in vitro* (44). This indicates that the miRNA-mediated regulation of butyric acid may be a potential regulator of SS tail adipose tissue growth.

MiRNAs have several classical molecular regulatory mechanisms, and in addition to binding to target mRNA transcripts *via* complementary base pairing, they can also target circRNAs to exert negative regulatory effects. Out of the 17,531 identified circRNAs, 198 DE circRNAs were screened in the current study. We found that these circRNAs were mainly up-regulated using expression trend enrichment analysis, which was the opposite of the main trend of miRNAs, which appeared to be negatively regulated. Therefore, we suggest that negative regulation between these molecules may be essential for the production of SS tail fat. To elucidate the regulatory relationship between miRNAs and circRNAs, we predicted the linkage between them. In the 30 vs. 6 M and 30 vs. 18 M comparative groups, it was observed that circRNA4175, circRNA1985, and circRNA382 bind to numerous miRNAs, which is noteworthy. In addition, we discovered that, among the three comparison groups, three miRNA families bind to the multiple circRNAs. They are miR-16 (chi-miR-16a-5p and oar-miR-16b_R+3) at 30 vs. 18 M, mmu-miR-6240-p5 at 18 vs. 6 M, and miR-27 (oar-miR-27a_R-1 and chi-miR-27b-3p) at 30 vs. 6 M. miR-27 is among the many miRNAs involved in cholesterol homeostasis and fatty acid metabolism. miR-27a and−27b were found to suppress adipocyte differentiation by regulating peroxisome proliferator-activated receptor γ (45). Previous research found that overexpression of miR-16a-5p can promote the expression of adipogenic marker genes as well as 3T3-L1 adipocyte differentiation by binding to the EPT1 gene (46). Current research on miR-6240 has focused on human heart disease, with one study suggesting that the downregulation of miR-6240 leads to an increase in white adipocyte markers (47). We also found that three circRNAs could bind to any two of these three miRNAs families, but only miR-27 could bind to all three. We therefore propose that the miR-27 family occupies a more central role in the interaction with circRNAs in the sheep tail adipose tissue.

In addition to regulating gene expression through competitive binding with miRNAs, circRNAs can also regulate the expression of their host genes (20). In the GO functional analysis of the circRNA host genes, we discovered that GTPase activator activity was enriched in both the 30 vs. 6 M and 30 vs. 18 M comparison groups. Recent studies have shown that GTPases play an important regulatory role in adipogenic differentiation (48, 49). Fatty acid beta-oxidation, which is crucial for the maintenance of thermogenesis, was among the significantly enriched GO terms for 18 vs. 6 M (50). KEGG pathway analysis suggested multiple adipose-related pathways were emphasized in our study. Among them, fatty acid metabolism was the only pathway enriched in all three groups, and host gene ACACA was enriched in these pathways. Combined with the findings of the circRNA host gene analysis (60 circRNAs from ACACA, Figure 1), we suggest that the majority of the circRNAs play a key metabolic role in tail fat.

Finally, we highlighted the potential regulatory mechanisms of ceRNAs in SS tail fat metabolism based on previous analysis of the expression profiles of miRNAs and circRNAs. In our study, we constructed two co-expression networks based on the mRNAs, miRNAs, and circRNAs identified in sheep fat tails. A total of 35 (down-up-down) and 19 (up-down-up) mRNA-miRNA-circRNA co-expression patterns were obtained. Herein, oar-miR-27a_R-1 was linked to multiple target genes, including TKT, ACSL4, and POSTN, which are involved in fatty acid metabolism and adipogenesis (50–52). As previously stated, miR-27 is a major regulator within adipose tissue, and miR-27a has been associated with lipid accumulation differences between intramuscular and subcutaneous adipose tissue in sheep (53). In the present study, miR-27a targeted multiple mRNAs and was shown to be regulated by up to four circRNAs. Therefore, we suggest that this miRNA may be a critical regulator of sheep tail fat development. Furthermore, oar-miR-29a binds to multiple mRNAs and circRNAs in the co-expression network. miR-29a was reported to associate with multiple biological processes, including lipid metabolism (54). In addition to TKT, miR-29a also targets to GPAM. GPAM was related to glycerolipid and glycerophospholipid metabolism, and its overexpression leads to the increase of triglyceride levels and lipid metabolism-related gene expression (55). Combined with the expression level of these two miRNAs, we suggest that oar-miR-29a and oar-miR-27a_R-1 play an important role in the ceRNA network. Therefore, we constructed the ceRNA interactions ACSL-oar-miR-27a_R-1-circRNA1985 and GPAM-oar-miR-29a-circRNA3539 using these two miRNAs as the core, and we successfully validated their interactions using dual luciferase gene reporter analyses. As a result, we verified two ceRNA expression networks and suggested multiple functions related to fat metabolism in sheep tail fat development. However, the involvement of these ceRNAs in sheep tail-fat metabolism requires further investigation. Overall, the investigation into the expression profiles of miRNAs and circRNAs at different developmental stages fills a research gaps in the study of the Sunite sheep tail fat metabolic mechanisms and provides new thoughts for future studies. For instance, these studies could focus on exploring the regulatory mechanisms of the novel miRNAs, the circRNAs that regulate their host genes, and the negative regulation of miRNAs, and on investigating more potential regulatory mechanisms for ceRNAs. The current results provide a theoretical basis for the identification of molecular markers related to sheep tail fat metabolism.

## Conclusions

In this study, we established miRNA and circRNA expression profiles to investigate the potential regulatory mechanisms underlying tail fat development in SS. At different growth stages, DE miRNAs may play a role in cell-to-cell interactions through target binding. The host genes of DE circRNAs were shown to be more involved in lipid and fatty acid metabolism. Based on the target prediction study, we created a miRNA-centered ceRNA regulatory network and filtered critical miRNAs for the validation of multiple target loci. Among them, we highlight the relationship pair of ACSL-oar-miR-27a_R-1-circRNA1985 and GPAM-oar-miR-29a-circRNA3539 ceRNAs centered on oar-miR-27a_R-1 and oar-miR-29a, revealing potential ceRNA networks involved in the regulation of tail fat development. Our findings highlight potential ceRNAs involved in sheep tail fat development and provide a theoretical basis for by-product utilization.

## Data availability statement

The datasets presented in this study can be found in online repositories. The names of the repository/repositories and accession number(s) can be found in the article/Supplementary material.

## Ethics statement

All experimental procedures were approved by the Animal Ethics Committee of the Inner Mongolia Agricultural University Animal Experimentation Area and followed the Chinese Animal Protection Law. Written informed consent was obtained from the owners for the participation of their animals in this study.

## Author contributions

XH and GB designed the study. XH, LC, YHa, and YHu performed the experiment. XH, RW, YY, and XQ analyzed the data. JW, LS, and GB provided ideas and suggestions. XH wrote the paper. All authors read and approved the final manuscript.

## Funding

This work was supported by the China Agriculture Research System of the MOF and the MARA (Grant No. CARS38) as well as the Inner Mongolia Autonomous Region Science and Technology Plan Project (2019–2022).

## Conflict of interest

The authors declare that the research was conducted in the absence of any commercial or financial relationships that could be construed as a potential conflict of interest.

## Publisher's note

All claims expressed in this article are solely those of the authors and do not necessarily represent those of their affiliated organizations, or those of the publisher, the editors and the reviewers. Any product that may be evaluated in this article, or claim that may be made by its manufacturer, is not guaranteed or endorsed by the publisher.

## Supplementary material

Supplementary Figure S1 GO function classification of DE miRNAs. **(A)** 30 vs. 6 M; **(B)** 30 vs. 18 M; **(C)** 18 vs. 6 M.

Supplementary Figure S2 GO function classification of DE circRNAs host genes. **(A)** 30 vs. 6 M; **(B)** 30 vs. 18 M; **(C)** 18 vs. 6 M.

Supplementary Table S1 qRT-PCR primer sequences.

Supplementary Table S2 The differentially expressed miRNA.

Supplementary Table S3 The differentially expressed circRNA.

Supplementary Table S4 The highly expressed DE miRNA in tail fat.
