# Supplementary figures and images for: MicroRNA and circular RNA profiling in the deposited fat tissue of Sunite sheep
Source: Front Vet Sci. 2022 Nov 4;9:954882. doi: 10.3389/fvets.2022.954882 (PMC9672515; doi:10.3389/fvets.2022.954882)

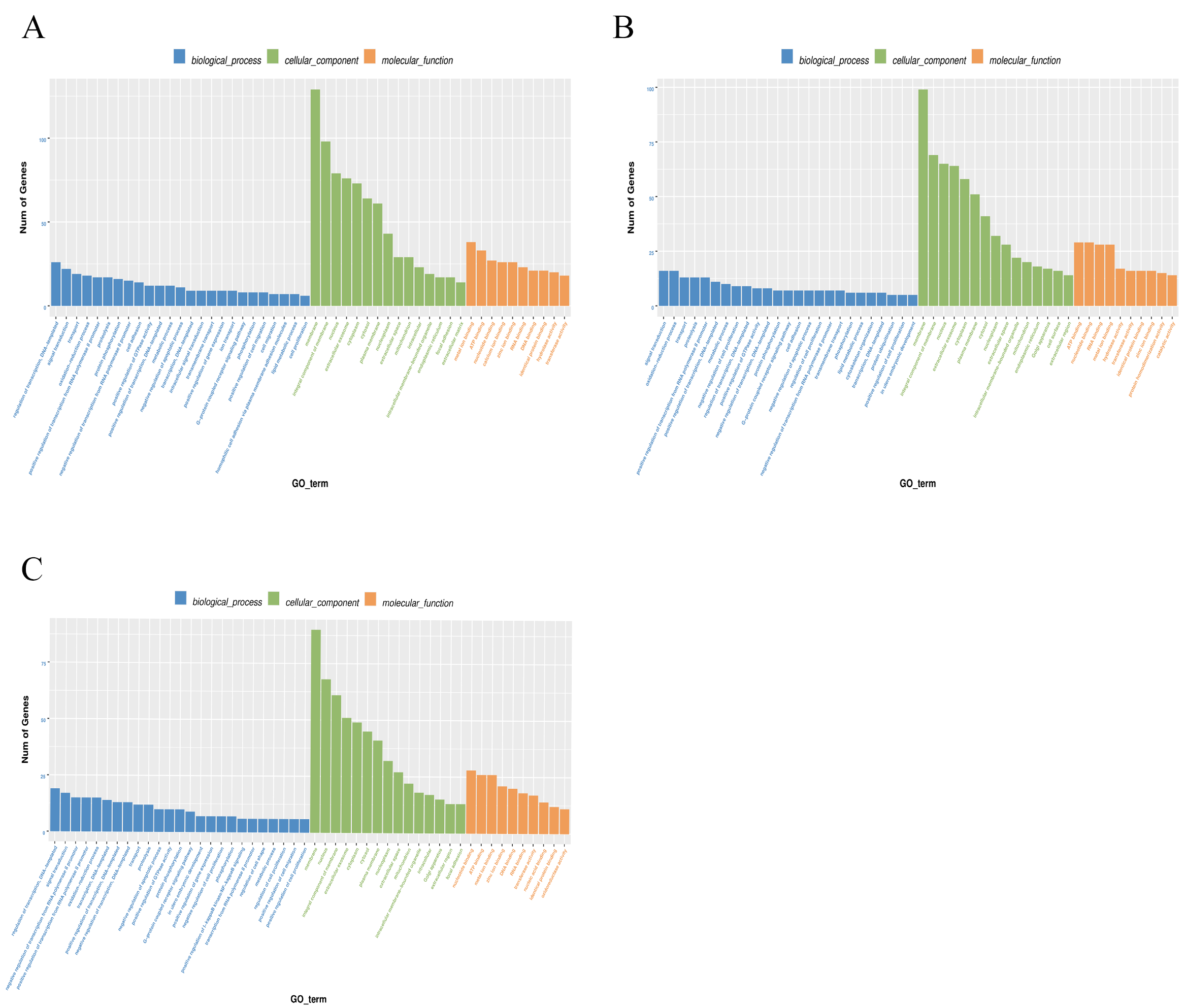

Supplement: Supplementary Figure S1 — GO function classification of DE miRNAs. (A) 30 vs. 6 M; (B) 30 vs. 18 M; (C) 18 vs. 6 M. [file Image_1.TIF]

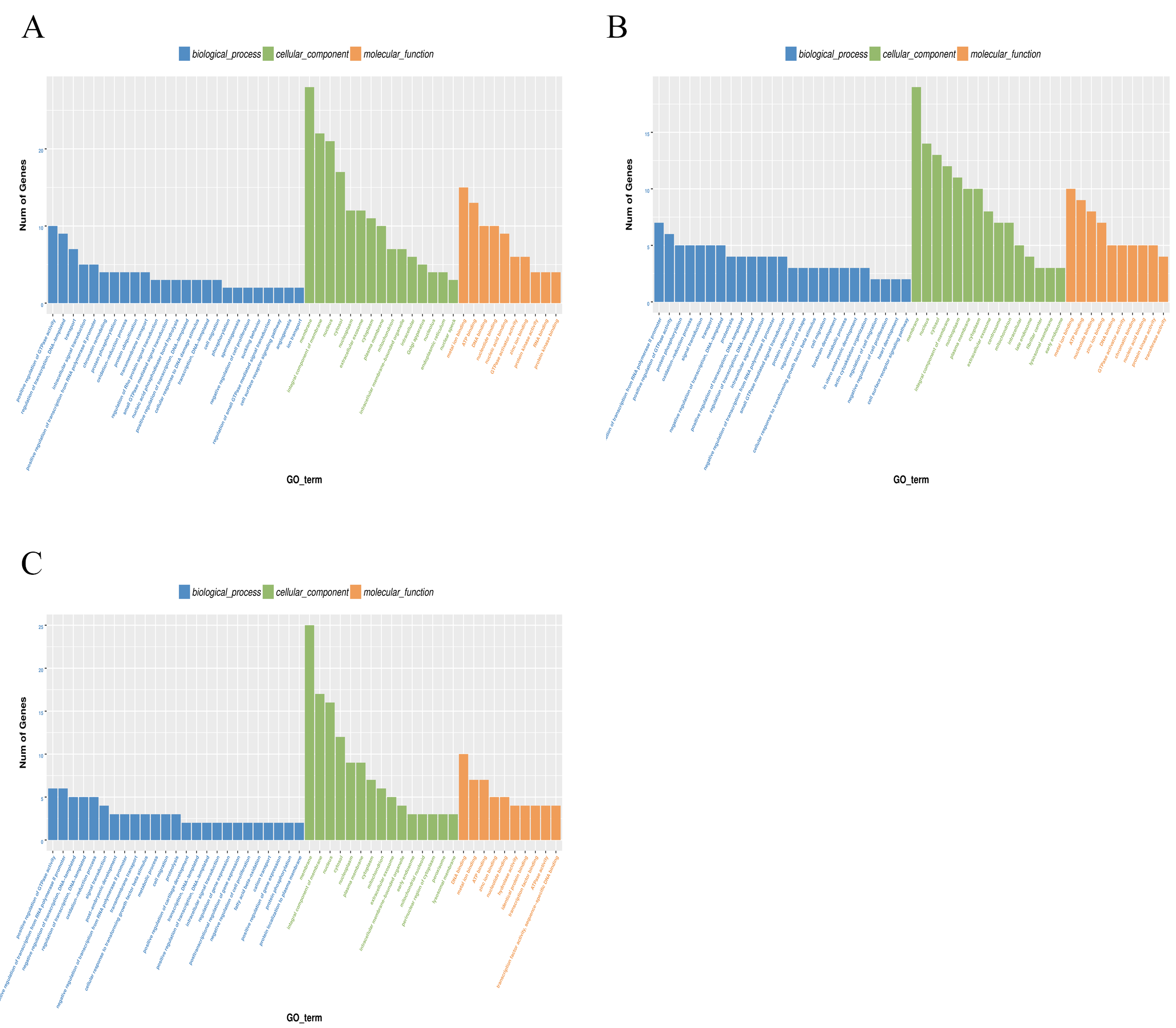

Supplement: Supplementary Figure S2 — GO function classification of DE circRNAs host genes. (A) 30 vs. 6 M; (B) 30 vs. 18 M; (C) 18 vs. 6 M. [file Image_2.TIF]
